# Evaluation of silver bio-functionality in a multicellular *in vitro* model: towards reduced animal usage in implant-associated infection research

**DOI:** 10.3389/fcimb.2023.1186936

**Published:** 2023-06-05

**Authors:** Leonardo Cecotto, Daphne A. C. Stapels, Kok P. M. van Kessel, Michiel Croes, Zeldali Lourens, H. Charles Vogely, Bart C. H. van der Wal, Jos A. G. van Strijp, Harrie Weinans, Saber Amin Yavari

**Affiliations:** ^1^ Department of Orthopedics, University Medical Center Utrecht, Utrecht, Netherlands; ^2^ Department of Medical Microbiology, University Medical Center Utrecht, Utrecht, Netherlands; ^3^ Infection Biology Group, Department of Biomolecular Health Sciences, Utrecht University, Utrecht, Netherlands; ^4^ Department of Biomechanical Engineering, Delft University of Technology, Delft, Netherlands; ^5^ Regenerative Medicine Centre Utrecht, Utrecht University, Utrecht, Netherlands

**Keywords:** biomaterial-related infection, antimicrobial, co-culture, cytotoxicity, immune response

## Abstract

**Background:**

Despite the extensive use of silver ions or nanoparticles in research related to preventing implant-associated infections (IAI), their use in clinical practice has been debated. This is because the strong antibacterial properties of silver are counterbalanced by adverse effects on host cells. One of the reasons for this may be the lack of comprehensive *in vitro* models that are capable of analyzing host-bacteria and host-host interactions.

**Methods and results:**

In this study, we tested silver efficacy through multicellular *in vitro* models involving macrophages (immune system), mesenchymal stem cells (MSCs, bone cells), and *S. aureus* (pathogen). Our model showed to be capable of identifying each element of culture as well as tracking the intracellular survival of bacteria. Furthermore, the model enabled to find a therapeutic window for silver ions (AgNO_3_) and silver nanoparticles (AgNPs) where the viability of host cells was not compromised, and the antibacterial properties of silver were maintained. While AgNO_3_ between 0.00017 and 0.017 µg/mL retained antibacterial properties, host cell viability was not affected. The multicellular model, however, demonstrated that those concentrations had no effect on the survival of *S. aureus*, inside or outside host cells. Similarly, treatment with 20 nm AgNPs did not influence the phagocytic and killing capacity of macrophages or prevent *S. aureus* from invading MSCs. Moreover, exposure to 100 nm AgNPs elicited an inflammatory response by host cells as detected by the increased production of TNF-α and IL-6. This was visible only when macrophages and MSCs were cultured together.

**Conclusions:**

Multicellular *in vitro* models such as the one used here that simulate complex *in vivo* scenarios can be used to screen other therapeutic compounds or antibacterial biomaterials without the need to use animals.

## 1 Introduction

Implant-related infections are one of the most frequent and severe complications associated with the use of biomaterials (Arciola et al., 2018). Despite best practice in medical and surgical management, infection occurs in approximately 5% of all operated orthopedic patients (Moriarty et al., 2016). The majority of orthopedic implant-associated infections (IAI) are caused by *Staphylococci*, in particular by *Staphyloccocus aureus* (Campoccia et al., 2006; Masters et al., 2022). This pathogen evolved multiple strategies to evade recognition and killing by our immune system and has developed resistance to commonly used antibiotics (Perret et al., 2012; Flannagan et al., 2015; de Jong et al., 2019; de Vor et al., 2020). Moreover, its ability to invade and survive within different host cells and tissues (Fraunholz and Sinha, 2012; Strobel et al., 2016; Horn et al., 2018) and infiltrate within the osteocyte canaliculi network (de Mesy Bentley et al., 2017) further complicates treatment. Altogether, these features make *S. aureus* one of the most challenging causes of IAI to treat with traditional antibacterial therapies.

Metallic silver (Ag) is a known, broad-spectrum antibacterial agent that was already used to treat infections before the introduction of antibiotics (Chopra, 2007). Over the years, numerous coating techniques have been developed to couple the antibacterial properties of silver to orthopedic implants (Amin Yavari et al., 2020; Djošić et al., 2021). Such Ag-coated implants have already been implemented in the clinic to decrease the incidence of infection after primary and revision surgeries, especially in oncologic patients who are susceptible to infections (Hardes et al., 2010; Wafa et al., 2015; Trovarelli et al., 2019; Diez-Escudero and Hailer, 2021; Fiore et al., 2021). However, the favorable antibacterial properties of silver are counterbalanced by adverse effects such as the skin blue coloring called argyria (Diez-Escudero and Hailer, 2021). Although most clinical studies do not report significant side effects, there is no consensus around the concentration at which silver may cause serious local or systemic damage (Eto et al., 2016; Diez-Escudero and Hailer, 2021; Fiore et al., 2021).

At a cellular level, many *in vitro* studies showed that silver exposure, both in free-ion (AgNO_3_) and nanoparticle (AgNP) form, correlates with DNA damage, increased production of inflammatory stimuli and reactive oxygen species, eventually leading to cell death (AshaRani et al., 2009; Park et al., 2010). However, whether AgNP effects are derived by the physical interaction with the nanoparticles, or the ions released from it is still under debate (Xiu et al., 2012; Durán et al., 2016; Liao et al., 2019). Nonetheless, nanoparticles might release lower concentrations of silver ions compared to AgNO_3_ (McQuillan et al., 2012; Ivask et al., 2014; Barros et al., 2019), therefore reducing silver toxicity. Moreover, as nanoparticles efficacy is shape-, size-, charge-, dose-, and time-dependent (Liao et al., 2019; Hamad et al., 2020), multiple studies tried to identify a therapeutic window where silver could retain its antibacterial activity while losing its toxicity to the host. Specifically, the optimal condition should not affect bone tissue and immune cells, which are key players involved in the post-operative (bone) healing process and control of infection around the implant.

Although the only place to start, simple *in vitro* studies are limited in the scope of their findings. For example, previous studies have shown that osteoblast viability, mesenchymal stem cell (MSC) proliferation and osteogenic differentiation were not negatively affected after prolonged incubation with silver at concentrations that still retain antibacterial properties (Qing et al., 2018; Rajendran et al., 2019; Souter et al., 2020). However, some signs of cell activation and toxicity were described at high silver concentrations (Hackenberg et al., 2011; Samberg et al., 2012; Pauksch et al., 2014), and other studies showed the negative impact of silver on osteoblast survival and MSCs osteogenic differentiation (Albers et al., 2013; Sengstock et al., 2014). In contrast to bone tissue, the viability of cells from the innate immune system is highly affected by silver. Micromolar concentrations of AgNO_3_ are already toxic to neutrophils (Croes et al., 2018), and macrophages could withstand exposure to silver for up to 24 h only at sub-antibacterial concentrations (Sarkar et al., 2015a). However, even such short exposure to silver negatively affected the metabolic activity of macrophages (Carrola et al., 2020) and their phagocytotic and bacterial killing properties did not improve (Sarkar et al., 2015b; Aurore et al., 2018). Although these studies provide useful insights into silver action against single types of host cells, they were incapable of verifying interactions between host cells and bacteria, and between host cell types that occur in the complex *in vivo* situation. Consequently, silver-coated implants with promising cytotoxicity and antibacterial properties *in vitro* generated contrasting results when tested in animal models (Chen et al., 2015; Croes et al., 2018).

Since the current *in vitro* models are not able to mimic a physiological environment, they consequently cannot predict *in vivo* behavior very well (Moriarty et al., 2014). Therefore, forced by regulatory authorities, medical device companies tend to undertake a vast amount of animal testing (Grainger et al., 2013). Not surprisingly, many of these animal trials are not conclusive due to variability between animals, highly scattered read-outs, and dead animals pre-/post-operation (Veening-Griffioen et al., 2019).

In order to better predict effectivity of antibacterial coating of implants and limit animal testing, we designed and built a multifaceted *in vitro* setup, primarily composed of a combination of host bone cells, bacteria, and immune cells. This model can precisely mimic the *in vivo* arena, test antibacterial properties and identify undesired foreign body responses to the developed implants. In our model, we chose to test the effectivity of two different forms of silver – AgNO_3_ or AgNP – due to their debatable effects on host-cell viability and bacterial survival. In our model, we co-cultured human bone marrow-derived MSCs, human monocyte-derived macrophages, and *S. aureus* in search of a therapeutic window for AgNO_3_ or AgNP that are used in implant coating, where neither the viability of bone nor immune cells was compromised. We here show that silver has its limitations as a therapeutic agent. This *in vitro* multicellular culture system represents a valid screening tool to predict the effects of various antibacterial compounds in an environment similar to the *in vivo* scenario without involving any animal testing.

## 2 Materials and methods

### 2.1 Human monocyte-derived macrophage culture

Blood from healthy human donors was supplied by the Dutch blood bank (Sanquin, Amsterdam, The Netherlands). Peripheral blood mononuclear cells (PBMCs) were isolated from buffy coats using Ficoll-Paque (Pharmacia, Uppsala, Sweden) density centrifugation. Monocytes were positively selected by magnetic-activated cell sorting (MACS) with anti-CD14 labelled microbeads (Miltenyi Biotec, 130050201) according to the manufacturer’s instructions.

Isolated monocytes were seeded in a 24-wells plate at a density of 3 x 10^5^ cells/well, except when stated otherwise. When monocytes were seeded on top of 13-mm diameter titanium disks (Alfa Aesar, 10385-HP), these were placed in the 24-wells plate before seeding. Monocytes were differentiated into macrophages by culturing for 7 days at 37°C, 5% CO_2_ in α-Minimum Essential Medium (α-MEM, Gibco Paisley, 22561021) supplemented with 10% (v/v) hyFBSclone fetal bovine serum (hyFBS, Biowest, HYCLSV30160), 100 U/mL penicillin- streptomycin (1% p/s, Gibco, 15140122), and 40 ng/mL human recombinant M-CSF (Peprotech, 300-25). Culture media was refreshed after 3-4 days.

### 2.2 Human mesenchymal stem cell culture

Mesenchymal stem cells (MSCs) were isolated from human bone marrow aspirates upon informed consent. The aspiration procedure was approved by the local medical research ethics committee, University Medical Center Utrecht, under the protocols METC 08-001/K and METC 07-125/C.

Aspirates were diluted in PBS, filtered through a 100 µm cell strainer and the mononuclear cell layer was collected after Ficoll-Paque density centrifugation. Approximately 2.5 x 10^5^ mononuclear cells were plated per cm² in MSC expansion medium consisting of α-MEM supplemented with 10% (v/v) heat-inactivated FBS (FBS, Biowest, S181H), 1% p/s, 0,2 mM L-ascorbic acid-2-phosphate (ASAP, Sigma-Aldrich, A8960) and incubated at 37°C, 5% CO_2_. Cells starting from passage 3 were used in the experimental setups.

### 2.3 Macrophage-MSC co-culture

To build the macrophage-MSC co-culture the following steps were adopted. Monocytes were seeded in 24-wells plates at a density of 3 x 10^5^ cells/well and differentiated into macrophages by culturing for 7 days. Meanwhile, MSCs were cultured to reach ~70% confluency until monocytes fully differentiated into macrophages. Then, MSCs were fluorescently labelled with celltrace violet (Invitrogen, C34557) diluted in Hanks balanced salt solution (HBSS) according to the manufacturer’s instructions for labelling adherent cells. After staining, MSCs were detached with 0,25% trypsin/EDTA (Gibco, 25200056) and re-seeded together with macrophages at a density of 1 x 10^5^ cells/well according to the experimental setup. Cells were allowed to adhere for about ~24 h before readouts started or bacteria were added, as explained below in the section “Multicellular infection model to study intracellular survival of bacteria”.

Cell seeding densities and culture plate format were adjusted according to the experimental setup. For instance, when measuring cytokine production, monocytes and MSCs were combined in a 96-well plate at a density of 1.5 and 0.5 x 10^5^ cells/well, respectively. Moreover, when the identification of single cell types was not relevant for the experimental setup, fluorescence labelling was omitted.

### 2.4 Bacterial culture

All experiments used GFP-labelled *Staphylococcus aureus* (kind gift from Prof. Simon Foster) and *Staphylococcus epidermidis* (kind gift from Prof. Leo Koenderman) were transformed with a GFP-expressing plasmid pCM29 to constitutively express GFP, as previously described (Boero et al., 2021). Bacteria were grown overnight in Todd-Hewitt broth (THB) with 10 ng/mL chloramphenicol to reach stationary phase.

### 2.5 Silver ions and nanoparticles

A solution of silver ions (AgNO_3_) was prepared by dissolving silver nitrate (Sigma-Aldrich, S6506) in ultrapure water. Commercially available 20 nm and 100 nm silver nanoparticles (AgNP) (Alfa Aesar, J67067 and J67099), were used. Before each experiment, both sizes of nanoparticles were pelleted by centrifugation for 30 min at 4°C with 17000 x g (20 nm AgNP) or 300 x g (100 nm AgNP). A stock solution of 80 µg/mL was prepared for each type of nanoparticle in ultrapure water. AgNP and AgNO_3_ were diluted to various concentrations in α-MEM with 10% FBS or in THB to test their effects on host or bacterial cells.

### 2.6 Effect of silver on cell viability and cytokine production

Monocytes and MSCs were seeded in a 96-wells plate at a density of 1.5 and 0.5 x 10^5^ cells/well, respectively. The same numbers of cells were combined in the co-culture, where macrophages were combined with MSCs as previously described.

Macrophages, MSCs, and the co-culture were incubated with fresh media containing 10 ng/mL LPS O111:B4 from *Escherichia coli* (Sigma-Aldrich), or various concentrations of AgNO_3_, 20 nm AgNP, and 100 nm AgNP. After 24 h stimulation, the culture medium was harvested to measure cytokine production and cells were processed for viability evaluation. When measuring cell viability, the culture medium was replaced by α-MEM with 10% FBS and 10% Alamar Blue solution prepared by dissolving Resazurin sodium salt (Sigma-Aldrich, R7017) in PBS. Then cells were incubated at 37°C for 2-3 h, in the dark. Next, the supernatant was transferred to a new plate and fluorescence was measured at 530-10/580-10 nm (Ex/Em) with a Clariostar plate reader (BMG labtech). Background fluorescence values were subtracted, and metabolic activity was normalized to the control sample. Production of cytokine TNF-α and IL-6 was measured in the collected supernatant by ELISA (Duoset, R&D Systems, DY210 and DY217B), according to the manufacturer’s instructions. ELISA values are expressed as fold-change over non-stimulated controls. Samples were analyzed in triplicates and experiments repeated three times with different monocytes and MSCs donors.

### 2.7 Direct antimicrobial properties of silver

The direct antimicrobial properties of silver were determined by measuring OD (600nm), combined with the broth microdilution method. Overnight bacterial culture was diluted in THB to reach a final inoculum of 5 x 10^5^ colony-forming units per mL (CFU/mL). In a flat-bottom 96-well plate, the bacterial suspension was mixed with AgNO_3_, 20 nm AgNPs, or 100 nm AgNP in equal parts in triplicates, with a final volume of 200 μL. Bacterial growth was monitored at 37°C by measuring OD (600nm) every 5 min, for a total time of 12 h, on a Clariostar plate reader with gentle shaking before each measurement. Then, bacterial suspensions were serially diluted and plated on Todd-Hewitt agar (THA), and colonies counted after overnight incubation at 37°C. Samples were analyzed in triplicates.

### 2.8 Multicellular infection model to study intracellular survival of bacteria

An overnight bacterial culture was diluted in α-MEM to reach a final concentration of 1 x 10^7^ CFU/mL. To mimic the *in vivo* situation, bacteria were incubated with 5% normal human serum (NHS) for 15 min at 37°C, which coats them with antibodies and complement (opsonization) to enable their uptake by immune cells. Serum was collected from blood obtained from healthy donors after informed consent, as previously described (Boero et al., 2021). Approval from the Medical Ethics Committee of the University Medical Center Utrecht was obtained (METC protocol 07-125/C approved on March 1, 2010).

Opsonized bacteria were added to the co-culture at various multiplicity of infection (MOI). To synchronize bacterial uptake, plates were centrifuged for 5 min with 110 x g at RT, and then incubated at 37°C, 5% CO_2_. To study intracellular bacterial survival, the cells were washed twice after 30 min of infection to remove free bacteria and cultured in media supplemented with 100 µg/mL gentamicin (Serva, 22185.02, to kill the bacteria) and 20 µg/mL lysostaphin (Bioconnect, MBS635842, to lyse the bacteria and lose the GFP signal) for 1 h. Afterwards, cells were washed twice and incubated in media with only 5 µg/mL gentamicin. All washing steps were performed with warm α-MEM. This treatment allows only intracellular bacteria to survive, as both gentamicin and lysostaphin are unable to penetrate mammalian cell membranes within short time periods (Hamza et al., 2013; Hamza and Li, 2014). To verify treatment efficacy in lysing bacteria, serum-opsonized *S. aureus* was incubated for 1 h at 37°C in α-MEM in presence of 100 µg/mL gentamicin and 20 µg/mL lysostaphin. Then, GFP expression was measured with a MACSquant VYB (Miltenyi Biotech) flow cytometer and data were analyzed with FlowJo (v.10.1., FlowJo LLC).

At the desired time points, co-culture samples were processed for flow cytometry analysis, microscopy observation, or quantification of intracellular bacteria *via* CFU count.

For flow cytometry, cells were detached by a combination of trypsin and eventually gentle scraping in 1 mM DPBS/EDTA if cells were still attached to the bottom of the culture plate. Cells were transferred to a 96-wells plate and stained with sytox orange dead cell stain for flow cytometry (Invitrogen, S34861) according to the manufacturer’s instructions. Samples were measured with a MACSquant VYB flow cytometer and data were analyzed with FlowJo. The gating strategy is summarized in **Supplementary Figure 1**. Briefly, a total of 10000 events were collected for each sample gated based on forward scatter (FSC) and side scatter (SSC) parameters. The two cell types were selected based on the signal of CellTrace violet. Non-infected samples from the sytox negative population were used to set GFP fluorescence baseline and define the proportion of infected, GFP-positive cells.

For confocal imaging, cells were collected as described for flow cytometry analysis and fixed in 1.5% paraformaldehyde. Cell membranes were stained with 3 µg/mL Alexa Fluor 647-conjugated Wheat Germ Agglutinin (WGA, Invitrogen, W32466) for 10 min at RT, on a shaking plate. Then, samples were transferred to CELLview slide (Greiner Bio-One, 543079) previously coated with poly-L-lysine (Sigma-Aldrich, P4707), and imaged on a Leica TCS SP5 microscope with a HCX PL AP CS 63x/1.40-0.60 OIL objective (Leica Microsystems). Images were adjusted for publication using Image J Fiji.

Finally, to quantify the number of intracellular bacteria, cells were lysed with 0.1% Triton X-100 and plated on THA plates in serial dilutions. Plates were incubated overnight at 37°C after which colonies were counted.

### 2.9 Multicellular infection model to study the effects of silver

A variation of this model was used to study silver effects. Briefly, cells were incubated for 24 h with different concentrations of AgNO_3_ and 20 nm AgNP before adding *S. aureus* at a MOI=10. Samples were analyzed by flow cytometry and CFU counting at 30 min and 4 h after infection. In this setup, when cells were incubated with silver, gentamicin and lysostaphin treatment was not employed for the 4 h time point. Samples were analyzed in triplicates and experiments repeated three times with different monocytes and MSCs donors.

### 2.10 Statistical analysis and graphics

GraphPad Prism 9 (version 9.3) was used to create the graphs and determine statistical significance *via* a two-way or one-way ANOVA, or t-test. *p*<0.05 was considered statistically significant. Illustrations were created with BioRender.com.

## 3 Results

### 3.1 Establishing a tunable, multicellular, *in vitro* model to study IAI

**Figure 1:**
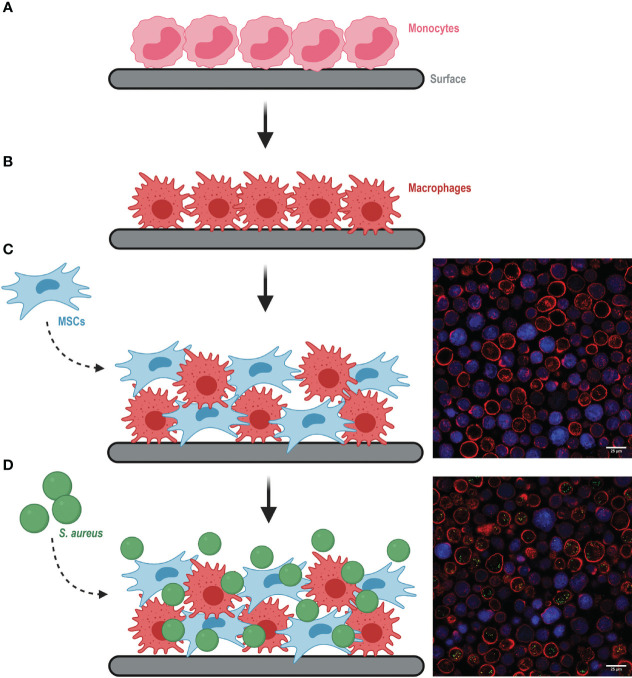
Schematic representation of building the multicellular *in vitro* model mimicking an implant-associated infection environment (images created with Biorender.com). **(A)** Monocytes were seeded on the selected surface and differentiated to macrophages **(B)** for 7 days. **(C)** Differentiated macrophages were re-seeded together with MSCs (left). Representative microscopy image of the co-culture (right), where MSCs were previously stained with CellTrace Violet (in blue) and all cell membranes were stained with Alexa Fluor 647-conjugated WGA (in red). **(D)** *S. aureus* was added to the co-culture (left). Representative image of the co-culture with intracellular *S. aureus* (green dots) (right).

To build a comprehensive, multicellular *in vitro* model that mimics the main players in IAI, we co-cultured primary MSC and monocyte-derived human macrophages as summarized in **Figure 1**. First, monocytes were isolated from human blood and seeded on a surface to differentiate into macrophages (**Figures 1A, B**). Cells were seeded either on a cell culture plate or on any biomaterial mimicking an implant. When comparing plastic with titanium, the surface itself seemed to have minimal impact on the phagocytic capacity of the macrophages (**Supplementary Figure 2A**). After differentiation, macrophages were re-seeded together with MSCs, which can differentiate into osteoblasts, to create an IAI-like environment (**Figure 1C**). In order to distinguish each element in the co-culture, we labeled at least one cell type before mixing with the second one. The co-culture was viable for up to several days, enabling the verification of cytotoxicity, modulation of cell functions, and antibacterial effects over a relatively long-time span.

The co-culture of macrophages and MSCs was then exposed to GFP-expressing bacteria (**Figure 1D**). We verified that this model could be used to study infections caused by different bacterial species relevant in the orthopedic field, such as *S. aureus* and *S. epidermidis*. Although macrophages were equally capable of associating with both bacterial species, we observed that MSCs were more susceptible to *S. aureus* than *S. epidermidis* infection (**Supplementary Figure 2B**). To assess the efficiency of antibacterial treatments in both macrophages and MSCs, all further experiments were conducted with *S. aureus*.

At the desired time points, the co-culture was processed for (confocal) microscopy (**Figure 1**), flow cytometry (**Supplementary Figure 2**), or bacterial enumeration *via* CFU plating. Flow cytometry offered the possibility to track intracellular bacterial survival per host-cell type over time by including gentamicin and lysostaphin treatment. These two compounds are not membrane-permeable and therefore selectively kill and lyse extracellular bacteria (Hamza et al., 2013; Hamza and Li, 2014). We confirmed by flow cytometry that the treatment efficiently lysed bacteria, as no GFP signal could be detected from *S. aureus* already after 1 h incubation with gentamicin and lysostaphin (**Supplementary Figure 2C**). Moreover, microscopy images confirmed that all GFP signal detected by the flow cytometer originated from intracellular bacteria and not from membrane-bound or extracellular *S. aureus* (**Figure 1D**). These results confirm the validity of the model we developed to study intracellular bacteria.

As expected, after 30 min of direct contact between bacteria and host cells, almost all macrophages had engulfed at least one *S. aureus* bacterium. In comparison, less than half of the MSCs population had engulfed bacteria. After 24 h, the proportion of infected macrophages was significantly reduced both when cultured alone, and in the co-culture. Interestingly, the proportion of infected MSCs was only reduced when cultured alone, but not in co-culture (**Supplementary Figure 2D**). This highlights distinct behaviors between cells cultured alone or together.

### 3.2 Finding a therapeutic window for silver treatment

We tested the validity of our co-culture model by using it to determine a possible therapeutic window for silver as an antibacterial treatment. For this purpose, silver must be at a concentration that is antibacterial, but not toxic to human cells. Therefore, we incubated *S. aureus*, macrophages, and MSCs with several concentrations of AgNO_3_, 20 nm AgNP, and 100 nm AgNP.

**Figure 2:**
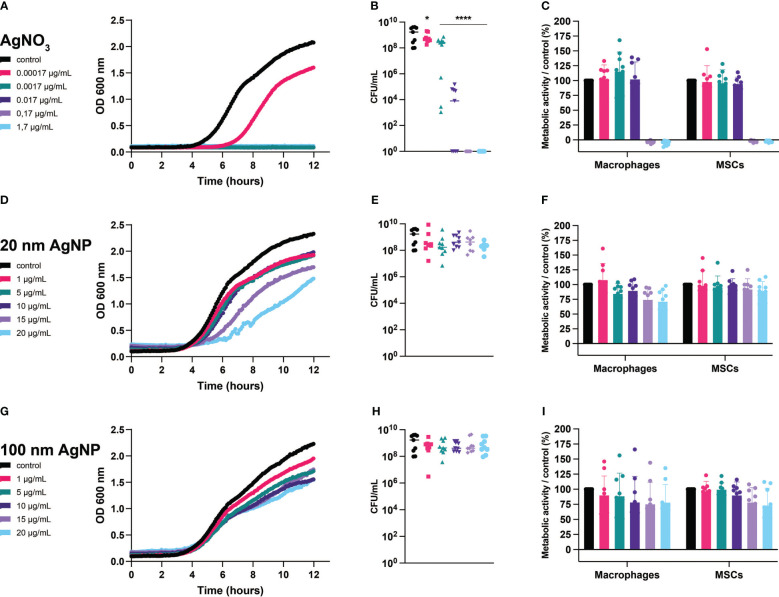
Evaluation of the effects of silver on *S. aureus*, macrophages, and MSCs. Bacteria and host cells were incubated with several concentrations of AgNO3 **(A–C)**, 20 nm AgNP **(D–F)**, and 100 nm AgNP **(G–I)**. Bacterial growth (graphs on the left) was monitored by measuring OD (600 nm) continuously during 12 h and the number of surviving bacteria was quantified by CFU count (graphs on the center). (n=9, from 3 independent experiments). Data for bacterial growth were plotted with mean only, and data for CFU count were transformed into log10 and represented as mean +/- SD. Statistical significance compared to control was determined *via* one-way ANOVA. *p<0,05; ****p<0,0001 on the log-transformed data. The metabolic activity of macrophages and MSCs (graphs on the right) was determined by alamar blue assay after 24 h incubation with silver formulations. Assay values were normalized to untreated control cells. Assay values were normalized to untreated control cells. (n=9, from 3 independent experiments). Data were represented as mean +/- SD.

We observed that at the lowest concentration tested, AgNO_3_ reduced bacterial growth and significantly decreased the number of viable *S. aureus*,while complete inhibition of growth and killing was achieved after exposure to AgNO_3_ concentrations higher than 0.0017 µg/mL (**Figures 2A, B**). Although bacterial growth speed was affected by increasing the concentrations of 20 nm AgNP (**Figure 2D**), no bacteriostatic or bactericidal effects were observed after incubation with 20 nm AgNP and 100 nm AgNP (**Figures 2D, E, G, H**).

On the other hand, AgNO_3_ completely abolished the metabolic activity of host cells starting at 0.017 µg/mL (**Figure 2C**), while exposure to AgNP, regardless of size or concentration, partially affected the metabolism of both cell types. For instance, concentrations of 20 nm AgNP higher than 1 µg/mL reduced the total metabolic activity of the macrophage population by up to ~25% (**Figure 2F**). Incubation with any concentration of 100 nm AgNP reduced the viability of macrophages, while the metabolic activity of MSCs was affected only at concentrations higher than 10 µg/mL (**Figure 2I**). Nonetheless, we exclude a toxic effect derived from exposure to nanoparticles as more than 75% of the total metabolic activity remained for both the macrophages and MSCs populations.

**Figure 3:**
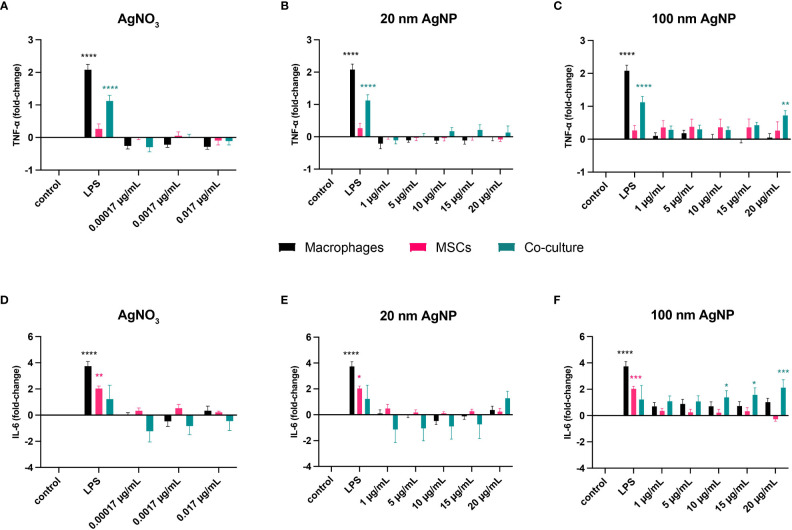
Evaluation of the inflammatory response caused by stimulation of host cells with non-toxic concentrations of silver. Macrophages, MSCs, and macrophages with MSCs (Co-culture) were incubated for 24 h with several concentrations of AgNO3, 20 nm AgNP, 100 nm AgNP, and 10 ng/mL LPS as positive control. Then, TNF-α **(A–C)** and IL-6 **(D–F)** levels in the supernatant were quantified by cytokine-specific ELISA and expressed as fold-change compared to control samples. (n=9, from 3 independent experiments). Data were transformed into 2-log and represented as mean +/- SEM. Statistical significance compared to control was determined *via* two-way ANOVA. ****p<0.0001; ***p<0.001; **p≤ 0.005; *p<0,05.

In addition, we assessed whether silver triggered an inflammatory response by measuring the secretion of TNF-α and IL-6. Overall, non-toxic silver concentrations did not elicit any inflammatory response in monocultured cells compared to control samples (**Figure 3**). However, from this assay we could observe the added value of co-culturing different cell types. Addition of MSCs reduced LPS-mediated activation of macrophages, as observed by decreased production of TNF-α. In addition, including immune cells reduced IL-6 production by MSCs. Moreover, exposure to 100 nm AgNP at 20 µg/mL and at concentrations higher than 5 µg/mL significantly increased TNF-α (**Figure 3C**) and IL-6 (**Figure 3F**) levels respectively in the co-culture.

### 3.3 Evaluation of the effects of silver on the multicellular *in vitro* model

**Figure 4:**
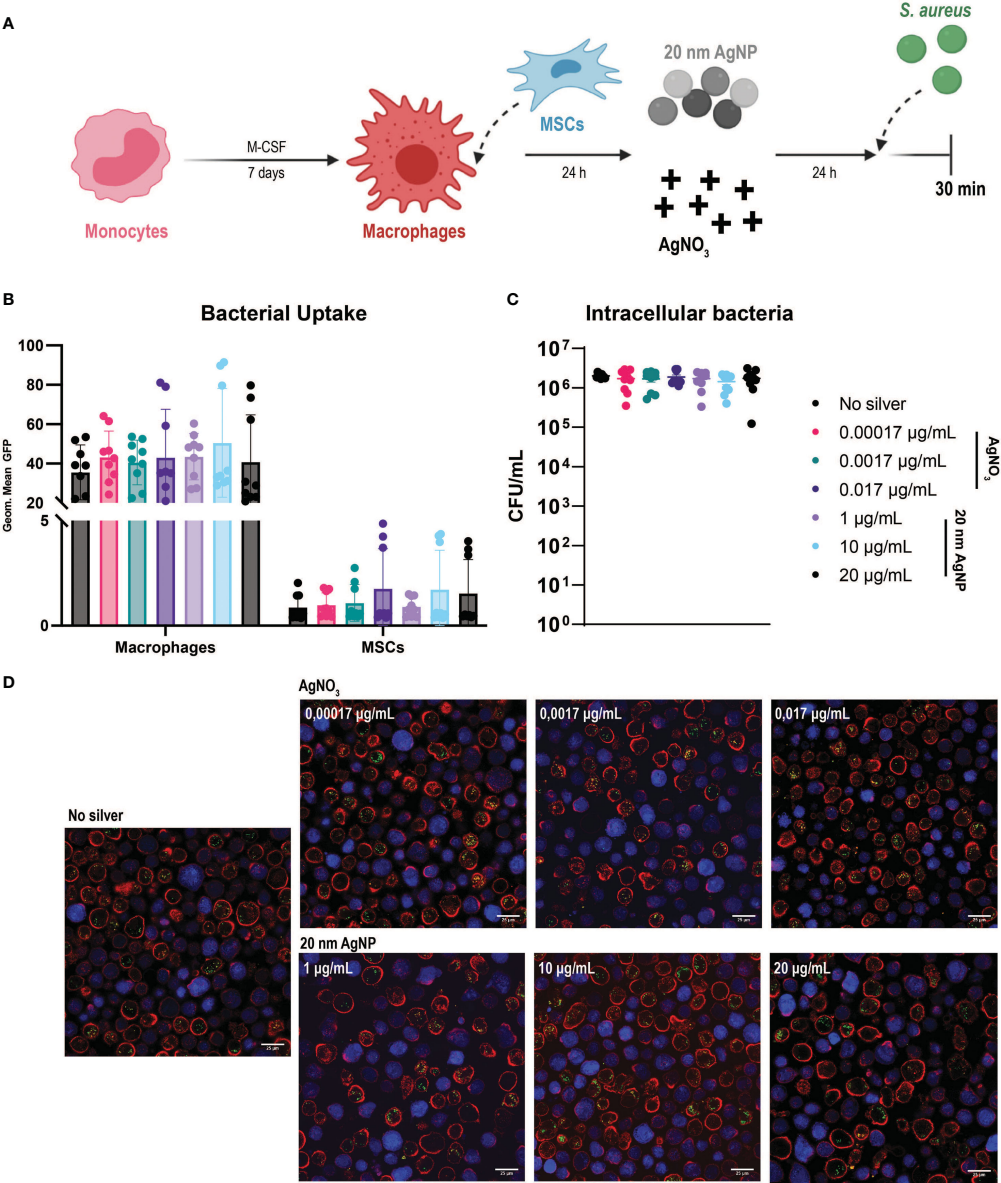
Evaluation of the effects of AgNO_3_ and 20 nm AgNP on the multicellular *in vitro* model **(A)** Schematic representation of the protocol used (images created with Biorender.com). Macrophages were combined with MSCs in culture and then incubated with AgNO_3_ and 20 nm AgNP for 24 h before infection with *S. aureus* for 30 min. **(B)** The amount of bacteria taken up by macrophages and MSCs is represented as the geometric mean of the GFP signal. **(C)** The number of intracellular bacteria was determined by counting CFU after cells lysis. (n = 9, from a total of 3 independent experiments). Data were represented as mean +/- SD. **(D)** Confocal imaging confirms flow cytometry observations. Macrophages (red), MSCs (blue), and *S. aureus* (green dots).

Once we established the effects of silver in simple *in vitro* models, we could further explore the consequences of its use in an IAI-like environment thanks to our multicellular model to study infection. Besides defining the antibacterial, non-toxic, and non-inflammatory concentrations of AgNO_3_ and AgNPs, we explored the impact of silver on the antibacterial functions of host cells (**Figure 4A**).

Due to their toxicity to host cells, we excluded AgNO_3_ at concentrations of 0.17 µg/mL and 1.7 µg/mL from the assays. Only three representative concentrations of 20 nm AgNP were selected, as similar cytotoxicity and antibacterial activity were observed. Analysis of samples by flow cytometry showed a remarkable shift in the side-scatter (SSC) values for macrophages in the co-culture when exposed to AgNP for 24 h (**Supplementary Figure 3A**). This increase in cellular granularity was concentration dependent and particularly evident in the presence of 100 nm AgNP. The effect was probably caused by the internalization of nanoparticles by macrophages, as shown by bright field microscopy (**Supplementary Figure 3B**). For this reason, 100 nm AgNPs were excluded from the analysis with the multicellular model.

Despite the previously observed antibacterial activity of AgNO_3_ (**Figures 2A, B**), this was not strong enough to counteract bacterial growth within the time frame tested in the multicellular model. In spite of the presence of AgNO_3_ or 20 nm AgNP, after 4 h incubation, the bacteria had already overtaken and killed most macrophages and MSCs in culture as observed by the increased amount of cells positive for sytox staining (**Supplementary Figure 4**).

Following *S. aureus* introduction into the co-culture, silver treatment did not influence bacterial uptake by host cells. We confirmed the presence of intracellular *S. aureus* in both cell types. As observed before, macrophages phagocytosed more bacteria than MSCs (**Figure 4B**). Depending on the donor, silver treatment had either no impact on phagocytosis or eventually led to an increased uptake of bacteria by both cell types. Moreover, exposure to silver did not reduce the amount of *S. aureus* able to survive intracellularly compared to the control samples (**Figure 4C**). Confocal microscopy imaging confirmed the flow cytometry results. Higher numbers of intracellular *S. aureus* were observed within macrophages than MSCs, while exposure to AgNO_3_ or AgNP did not influence bacterial uptake (**Figure 4D**).

## 4 Discussion

Despite the long-known antibacterial efficacy of silver, its clinical use has lagged. One cause might be ascribed to the lack of reliable *in vitro* models to test silver’s efficacy. Another limitation might be related to the need to identify a therapeutic window where pathogens are killed without affecting the viability of host cells. In this work, we developed a multicellular *in vitro* model that mimics an IAI-like scenario to investigate the efficacy of silver as a therapeutic treatment.

Traditionally, the effect of silver on bacteria or host cells was investigated separately, ignoring cell-cell interactions. This often rushed towards the identification of silver concentrations that were antibacterial and non-toxic for host cells (Priebe et al., 2017; Huang et al., 2022; Kuo et al., 2022; Selvamani et al., 2022) without addressing interactions between bacteria and host cells, and between host cell types. Although they did not address the antibacterial properties of silver, other research groups have shown that the effectiveness of novel treatments was different when assessed on cells cultured alone or in the presence of bacteria (Zaatreh et al., 2016; Luan et al., 2020). Similarly, encouraging results have been achieved after tests run on monocultures *in vitro*, but generated opposite outcomes when these treatments were tested in animal models (Chen et al., 2015; Tuchscherr et al., 2016). In a previous study, we showed that AgNP-coated surfaces completely killed *S. aureus in vitro*, while they did not exert any bactericidal effect *in vivo*. Moreover, the presence of coated implants worsened the healing process. Afterward, we learnt that these side effects derived from previously undetected Ag toxic levels against immune cells (Croes et al., 2018). Therefore, to improve the reliability of our *in vitro* tests, we designed a comprehensive multicellular model where cell-cell interactions can be investigated.

The interaction between MSCs and macrophages have already been showed to improve osteogenic differentiation of MSCs (Nicolaidou et al., 2012; Loi et al., 2016), resolution of infection (Krasnodembskaya et al., 2012; Johnson et al., 2017a; Chow et al., 2020) and inflammation (Lu et al., 2021). We also assessed the additive value of culturing macrophages and MSCs together. In fact, in the presence of LPS, cells in co-culture secreted a lower amount of cytokines compared to single cells (**Figure 3**). On the other hand, although immune cells captured most of the invading pathogens, a small fraction of MSCs was still infiltrated by *S. aureus*, *via* a process that could have been either active or passive (Fraunholz and Sinha, 2012; Josse et al., 2015). In co-culture, 24 h after infection, we observed a reduction of intracellular bacteria only within macrophages, with no changes in MSCs (**Supplementary Figure 2D**). While this could be explained in immune cells by the activation of their bactericidal activity (Pidwill et al., 2021), further studies are needed to understand the antibacterial mechanisms activated in MSCs when seeded alone but not in co-culture. Nonetheless, we have shown that our multicellular model allows us to identify each element involved in culture as well as track the intracellular survival of bacteria over time. As cells could also be seeded on different surfaces (**Supplementary Figure 2A**), this model can be extended to study the impact of new biomaterials and/or coatings on host cells and bacteria. Here, we used the model as a preliminary screening tool for finding the optimal silver concentrations to include in antibacterial coatings for orthopedic implants.

There are several reasons to favor silver formulations as nanoparticles rather than free ions. Use of higher-sized nanoparticles with a larger surface area is thought to reduce the amount of silver ions released, and therefore reduce AgNPs toxicity against host cells (McQuillan et al., 2012; Ivask et al., 2014; Barros et al., 2019). Accordingly, nanoparticles with larger sizes need to be used at higher concentrations to achieve similar efficacy to AgNO_3_ (Albers et al., 2013; Lu et al., 2013; Pazos-Ortiz et al., 2017; Qais et al., 2019). Besides a reduction in cytotoxicity, it should be considered that continuous uptake of non-degradable nanoparticles by host cells eventually leads to alterations in cell shape and morphology, as shown by our flow cytometry analysis (**Supplementary Figure 3**). Moreover, AshaRani et al., showed that nanoparticle uptake *in vitro* did not correlate with substantial cell death, even after incubation with higher concentrations of AgNP with a size distribution lower than 20 nm, but forced macrophages into a state of metabolic arrest (AshaRani et al., 2009). Instead, nanoparticle dimensions affected *in vivo* clearance and tissue accumulation, with the risk that particles larger than 40 nm reside indefinitely within the body (Gustafson et al., 2015). Furthermore, continuous exposure to nanoparticles might cause the onset of a local inflammatory response in the long term with detrimental consequences for implant-tissue integration (Zreiqat et al., 2003). According to our results, AgNPs were less toxic to host cells and bacteria than AgNO_3_ (**Figure 2**). Although we observed only a slight reduction in metabolically active macrophages and MSCs after exposure to AgNPs, previous studies suggest that use of higher concentrations might have induced cytotoxic effects on host cells as well (Arai et al., 2015; Sarkar et al., 2015a; Aurore et al., 2018; Carrola et al., 2020). For instance, the onset of harmful effects on host cells was evident from the inflammatory response caused by 100 nm AgNP (**Figures 3C, F**). Interestingly, this side effect was detected only with the use of the co-culture model rather than with single cell assays. Furthermore, treatment with neither 20 nm AgNP or AgNO_3_ reduced the number of intracellular bacteria found in macrophages and MSCs (**Figure 4**).

The multicellular model we used only allowed us to assess silver’s efficacy for a limited period of time due to the fact that bacteria grow faster than host cells. While AgNO_3_ displayed bacteriostatic and bactericidal effects after 12 h (**Figures 2A, B**), the same concentrations failed to inhibit *S. aureus* growth after 4 h in the multicellular model (**Supplementary Figure 4**). This limited our study to bacteria that survive intracellularly, while this multicellular model might be used to assess the efficacy of treatment on both extracellular and intracellular bacteria. The observed change in the efficacy of silver might be caused by the assay conditions *in vitro*. For instance, aerobic or anaerobic culture conditions (Yang et al., 2013), aggregation of nanoparticles (Brennan et al., 2015) or binding of silver ions to serum proteins (Gosheger et al., 2004; Shahabadi et al., 2012) could negatively impact the bio-functionality of silver. Our study might be affected by this, since different types of media were used to assess silver toxicity against *S. aureus*, macrophages, and MSCs. Moreover, silver exposure may even affect the response of macrophages to invading pathogens. For instance, Sarkar et al., showed that AgNP-treated macrophages had a reduced cytokine response and activation following *Mycobacterium tuberculosis* infection (Sarkar et al., 2015a). On the other hand, other research groups did not observe any positive impact on phagocytosis or oxidative burst in innate immune cells after treatment with silver (Liu et al., 2013; Haase et al., 2014; Huang et al., 2021).

Apart from testing the efficacy of silver as an antibacterial agent, this model could be used as an *in vitro* screening tool for several other therapeutic compounds (Cecotto et al., 2022) or antibacterial coatings. By developing culture models in which host-host and host-bacteria cells interact, we may gain greater insight into how implant surface features influence these cells, which has been investigated only on single elements so far (Leach and Whitehead, 2018; Liu and Segura, 2020; Zhu et al., 2021; Monteiro et al., 2022; Tan et al., 2022). In fact, this model could be adapted to study different scenarios of IAI. In accordance with the race for the surface theory (Gristina et al., 1989), bacterial colonization of the implant surface may prevent host cells adhesion and subsequently implant-tissue integration. Accordingly, Luan et al., showed that variations in gold nanoparticle-coatings were able to modulate macrophages functions and bone cells adhesion according to the presence or absence of bacteria on the coated surface (Luan et al., 2020). However, these and other studies, including ours, were performed under static conditions *in vitro*. By adding a flow system that simulates *in vivo*-like conditions such as shear stress and exchange of nutrients and molecules among cells in culture, outcomes can be closer to a real IAI scenario (Subbiahdoss et al., 2011; Zhao et al., 2014; da Silva Domingues et al., 2015). Finally, these multicellular models could include different cell types involved in IAI, such as osteocytes, osteoclasts, or cells from the bone marrow. Neutrophils and macrophages, for example, form the first line of defense against invading pathogens (Rosales, 2018; Rosowski, 2020; Pidwill et al., 2021). Despite previous reports about neutrophil interactions with single cell types and different biomaterials (Silva, 2010; Brandau et al., 2014; Jhunjhunwala, 2018; Abaricia et al., 2020; Wesdorp et al., 2023), little is known about their role in complex *in vitro* models that mimic the conditions of IAI.

## 5 Conclusion

Thanks to our multicellular model combining two host-cell types, bacteria, and an implant surface, we can predict the possible benefits and pitfalls derived from the use of silver as an antibacterial agent. Although our monoculture assays suggested the existence of a therapeutic window for the use of AgNO_3_ between 0.00017 and 0.017 µg/mL, our multicellular model revealed that neither the extracellular nor intracellular survival of *S. aureus* was affected. Moreover, we found that uptake of 20 nm AgNP had no impact on metabolic activity, phagocytic and killing capacities of macrophages, whereas increasing the size of the nanoparticles caused an inflammatory response that was only detectable when macrophages and MSCs were cultured together. By developing a model that accounts for the interactions among host cells and bacteria, *in vitro* screening tests can better simulate the complexity of *in vivo* models and better predict treatment outcomes.

## Data availability statement

The raw data supporting the conclusions of this article will be made available by the authors, without undue reservation.

## Author contributions

LC, DS, HW and SA contributed to the conception and design of the study. LC, DS, ZL performed the experiments. LC, DS, and SA wrote the first draft of the manuscript. All authors contributed to the article and approved the submitted version.
