# Supplementary material for: Evaluation of silver bio-functionality in a multicellular in vitro model: towards reduced animal usage in implant-associated infection research
Source: Front Cell Infect Microbiol. 2023 Jun 5;13:1186936. doi: 10.3389/fcimb.2023.1186936 (PMC10277478; doi:10.3389/fcimb.2023.1186936)
Supplement: Supplementary file 1 [file DataSheet_1.pdf]

Supplementary Material

Evaluation of silver bio-functionality in a multicellular *in vitro* model: towards reduced animal usage in implant-associated infection research

Leonardo Cecotto, Daphne A.C. Stapes, Kok P.M. van Kessel, Michiel Croes, Zeldali Lourens, H. Charles Vogely, Bart C. H. van der Wal, Harrie H. Weinans, Jos A.G. van Strijp, Saber Amin Yavari

*** Correspondence:** Harrie Weinans, h.h.weinans@umcutrecht.nl

1 Supplementary Figures

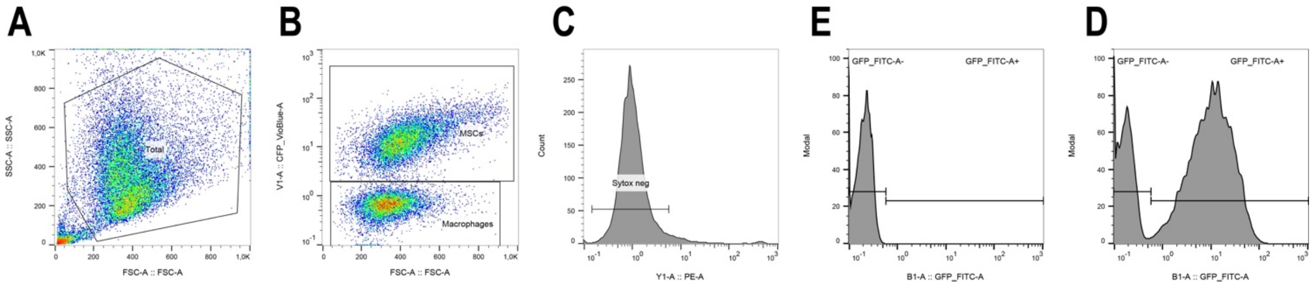

Supplementary Figure 1. Flow cytometry phagocytosis gating strategy. (**A**) Selection of the total cells population (total gate) in the linear FSC and SSC. (**B**) Selection of the two cell types in the linear FSC and fluorescence at 405 nm wavelength. (**C**) Selection of sytox negative cells within each cell type population. (**D**) Histogram setting GFP fluorescence baseline for non-infected cells. (**E**) Proportion of non-infected (left peak) and infected (right peak) cells.

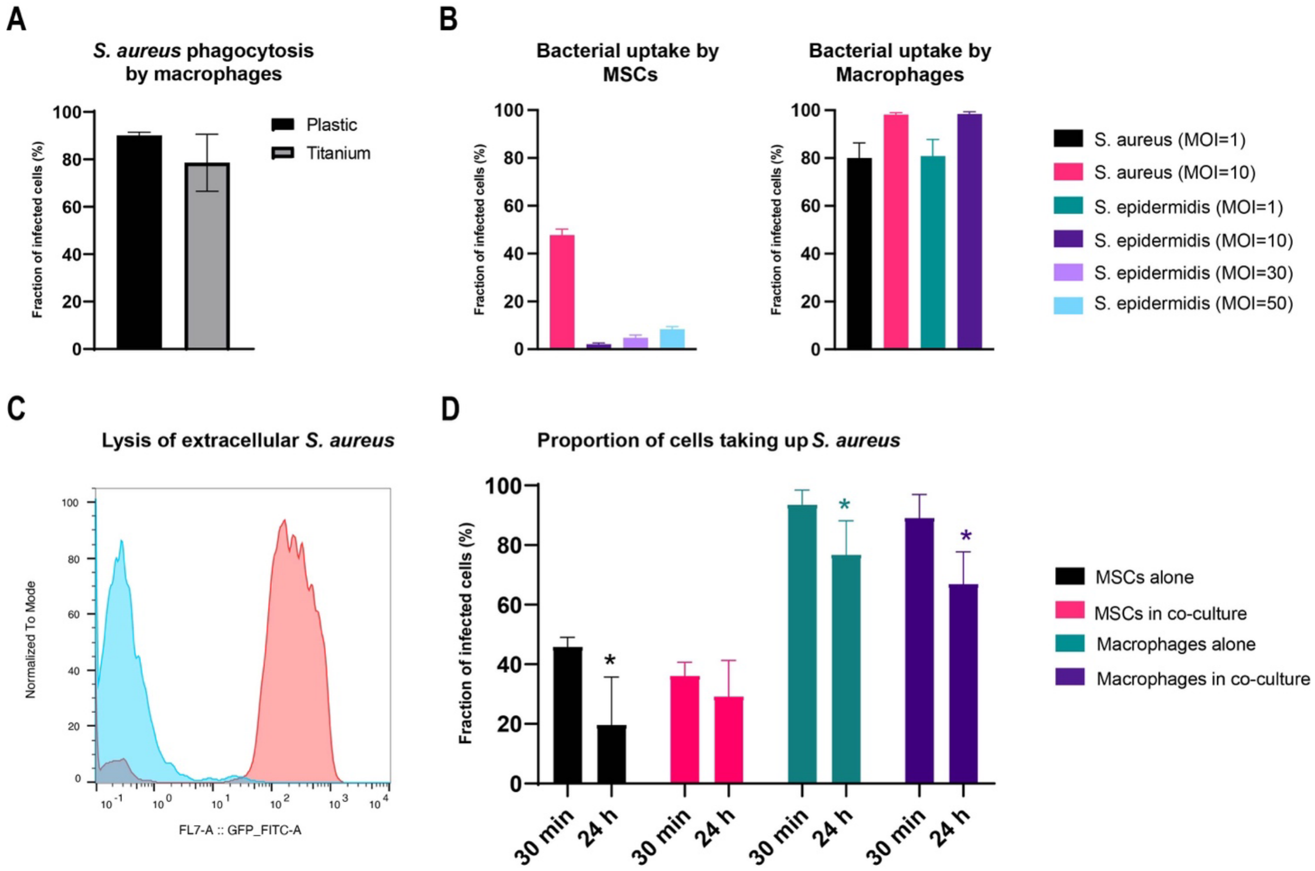

Supplementary Figure 2. Different applications of the multicellular model according to the research question, analyzed by flow cytometry. (**A**) Monocytes were seeded either on a culture plate (plastic) or on a titanium disk and differentiated to macrophages. Next, cells were exposed to *S. aureus* for 30 min and the proportion of cells that had ingested at least 1 bacterium was determined. (**B**) MSCs and macrophages were seeded alone and then exposed for 30 min at increasing MOIs of S. aureus and *S. epidermidis* to determine the strain dependent-uptake for each cell type. Proportion of cells that ingested at least 1 bacterium is shown. (**C**) Serum-opsonized *S. aureus* was incubated at 37ºC in presence of 100 µg/mL gentamicin and 20 µg/mL lysostaphin. After 1 h incubation, bacterial GFP signal was measured by flow cytometry and the histograms of control (red) and treated (blue) bacteria were compared. (**D**) Macrophages and MSCs were seeded alone or in combination with each other and infected with *S. aureus*. The proportion of cells with intracellular bacteria was calculated at 30 min and 24 h after infection. Data were represented as mean +/- SD. Statistical significance compared to 30 min was determined via t-test, *p<0,01.

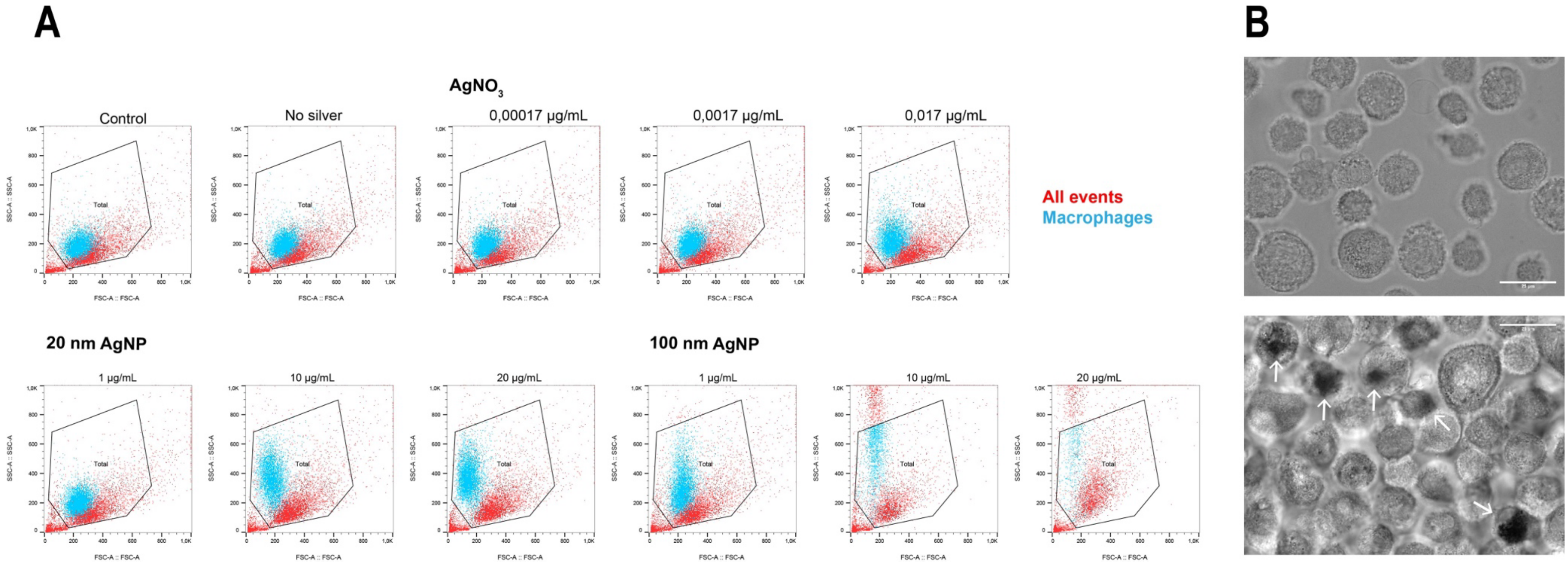

Supplementary Figure 3. Influence of nanoparticles uptake in macrophages. (**A**) Dot plots from co-culture samples showing the distribution in the FSC and SSC axis of the macrophage population (blue) within all the events recorded by the flow cytometer (red). (**A**) Bright field images showing the change in morphology of macrophages before (upper image) and after (lower image) 24 h incubation with AgNP 20 nm at 20 µg/mL. White arrows pointing at intracellular nanoparticles.

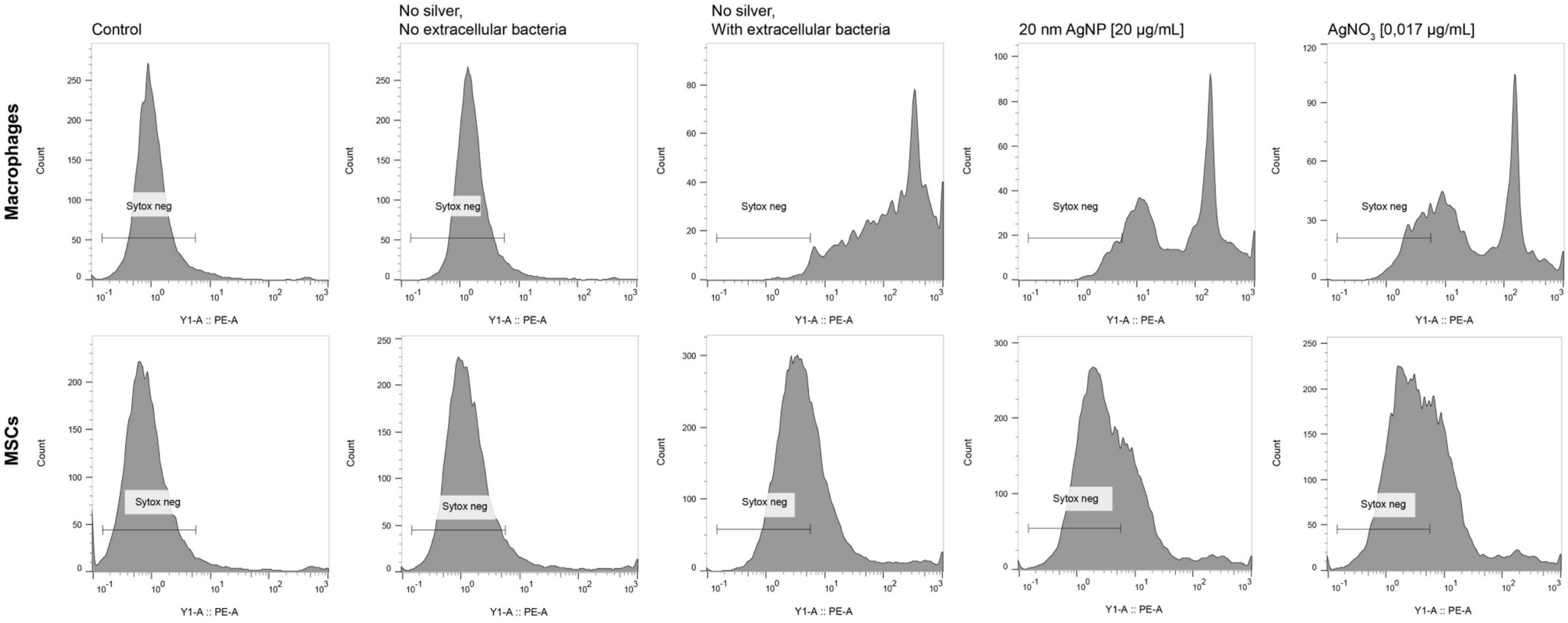

Supplementary Figure 4. Selection of sytox negative macrophages (upper row) and MSCs (lower row) after incubation for 4 h with *S. aureus*.
